# Supplementary material for: How research data deliver non-academic impacts: A secondary analysis of UK Research Excellence Framework impact case studies
Source: PLoS One. 2022 Mar 10;17(3):e0264914. doi: 10.1371/journal.pone.0264914 (PMC8912237; doi:10.1371/journal.pone.0264914)
Supplement: Attachment — Submitted filename: Response to Reviewers (Jensen et al.).docx [file pone.0264914.s001.docx]

**Resubmission (rebuttal) Letter**

Response to Reviewers

Editor

PLOS ONE

31 July 2021

Dear PLOS ONE Team,

I hope this letter finds you well. I am writing to you on behalf of myself and my co-authors again regarding our manuscript “How research data delivers non-academic impacts: A secondary analysis of UK Research Excellence Framework impact case studies”.

The comments from the reviewers have been pasted in below with responses in blue.

The existing literature about non-academic impact in relation to REF should be discussed. A quick search in Web of Science [ ts=(REF and ("societal impact" or "non-academic impact") ) ] reveals previous studies that would be suitable for inclusion in such a short literature overview.

Additional literature has been integrated into the introduction and discussion, focusing on other recent analyses of REF2014 case studies, and the inclusion of impact in REF.

Lines 159 and 160 refer to Krippendorff’s Alpha as "Kalpha". However, Table 2 uses "Alpha" as a column header. That is somewhat confusing.

Amended to use “Kalpha”

Lines 181-183 contain duplicated phrases ("... types of impacts that are most commonly linked most commonly linked to research data. ... revealed a set of different types of impact different types of impact ...") and do not have a proper ending. This paragraph has to be rephrased.

Amended.

Some table numbers seem to be wrong. Please check the cross-referencing in lines 192, 196, 233, and 238.

All table numbers amended

Table 3 defines eleven types of impact but Table 4 contains only seven types of impact plus a type named "other". Are the remaining types grouped into "other"? If so, this should be explained in the text. This new group "other" seems rather redundant as there has already been another group with "Other Kind of General Public Impact"

Amended as “Remaining”

Line 201 has a duplicated explanation of the abbreviation WRQoL, once in line and once in the footnote. One explanation is enough.

Footnote removed.

On Table 6, the impact developments "Mobile App" and "Unclear/Uncertain are missing in comparison with Table 5 without any explanation.

Amended to remove unclear/uncertain as a low interest category from Table 5 and add mobile app category to Table 6 to align the two tables.

After adding the short literature overview mentioned above, the authors should highlight their contributions beyond the knowledge from prior literature.

The conclusion now builds on the additional literature to make a clearer statement of the study’s original contribution.

I appreciate that the authors are providing data and code. However, the manuscript should include more information about the used program, i.e., which version of R was used, and which packages were loaded? The code loads a long list of packages. These packages have recommended citations that should be included in the manuscript, see for example: citation('ggplot2').

Amended with references to all packages used.

Reviewer #2:

The general research question and how the methodological approach is aligned to the research question seems a bit unclear: is it about (1) the impact of providing research data, or (2) the impact of using research data (or something else). The methodological approach does not seem to be able to distinguish these two approaches. If (2) should be the focus of this paper, I am not sure whether focusing on the TF-IDF values above the 3rd quartile (ll. 140-143) is necessary at all (if "data" is mentioned, the case is very likely to make use of some sort of data; what does it mean if the term "data" is mentioned many times in a submission, compared to only once?).

The answer is (1) because the source of these data is REF impact case studies which are generated by/about researchers (in this case researchers who provided research data as a means of achieving impact). Further context has been added to the methods section to help to clarify.

98: "3" -> "3*"

Amended.

Section 2: A more detailed description of the REF data would be helpful, e.g.: What entities are evaluated in the REF2014 (Papers, Projects, …)? Who evaluates them? What do the *-ratings mean? From which disciplines are the cases / how does the distribution across disciplines look like (if possible)? How long are the narratives / is there a minimum/maximum number of words? This could be placed in an additional subsection in section 2. Also the paragraph from line 167 to line 173 could be moved in this new subsection.

Additional details have been added to section 2

127-129: what does "are … featured prominently" mean here, can this be specified (e.g., if the share of words indicating data-related case exceeds a certain threshold, the case is selected)?

Amended “featured prominently” removed.

140-142: "… are within the third quartile": Should this be "… are above the third quartile"? The third quartile is a particular number, so a TF-IDF cannot be within the third quartile.

Amended to “above the third quartile”

141-143: For the cases identified as data-related, a manual check on what role research data actually play in these cases could be included here. This may also help to address my first comment.

“A manual review of these 148 cases were conducted to ensure their relevance” was added to the text before table 1.

144: Here, the authors could mention (in the text) the number of cases remaining after excluding all cases not having 3* or 4* ratings, and also insert a reference to table 1.

Amended in main text to explicitly mention the number of cases

147-151: A short description of the content analysis (how the categories were defined, how they were assigned to the cases) would help the reader to understand the results and also reliability analysis.

Amended in main text to extend the explanation of the content analysis process, including inter-coder reliability analysis

159: Are the 52 units all of the units that occur in the data?

It has been clarified in the main text that the 52 units referenced comprise the sub-sample used for the inter-coder reliability analysis

Table 2: Where are these categories used? They seem to be similar to the categories presented in Table 3 and Table 5, but they are not identical.

This table was confusing because it included a mix of different variable levels. It has now been edited to show where analysis is at the overall variable level versus specific categories under that overall variable. In addition, there were variables reflected in the table that are not included in the results presented in this paper. These have been deleted.

181-183: There seem to be some typos in this paragraph.

Amended

Section 3.1: Some additional information (and a reference to the supplementary material) on the impact types presented in the table and how they were defined / assigned to the cases would be helpful to understand the result and to assess their objectivity. In particular, the following questions should be answered: The categories in Table 3 don't occur in Table 2; are

Additional detail about the process of developing the categories / analytic framework has been added to the methods section.

Table 2 has been edited to more clearly show what was included in the inter-coder reliability analysis

Why are the two categories "Government Spending / Efficiency Impact" and "Other Government / Policy Impact" listed separately in the table, while they are merged in lines 189-190?

These are simply combined to provide an overall summary for the reader. The underpinning analysis was at the more specific level.

A problem of these impact types may be that they have different levels of specificity (e.g., "Practice Impact" is a very general impact type that may apply to cases from various disciplines, i.e. it is no surprise that it is assigned to many cases; in contrast, "Public Health Impact" or "Environmental Impact" are more specific, since they refer to a particular discipline).

The focus here is on the nature of the impact. This is the reasons for separating these in the way they are. Given they have proven to be robust in their ability to applied objectively (demonstrated by the inter-coder reliability analysis), we don't see a reason to adjust the categories and redo this entire analysis from scratch (what would be required if the categories were shifted at this stage). We have conducted a separate analysis that will be presented elsewhere that looks at the nature of the beneficiaries of impact.

188-192: Can multiple impact types be assigned to one case, or exactly one impact type for each case?

Yes. This has been made explicit in the main text now:

More than one category could be allocated to each impact case study, if applicable.

192 & 196: "Table 2" -> should this be Table 4?

Amended

201-202: "Work-Related Quality of Life Scale(WRQoL) scale" -> "Work-Related Quality of Life (WRQoL) scale"

“Scale” removed

Section 3.2: As in section 3.1, some information on how the categories presented in Table 5 were determined and assigned to the cases would be helpful to understand (see the comments on section 3.1). Also the specificity of the categories seems also be problematic here and should be addressed.

This comment overlaps with a previous one, and has been addressed there.

238: "Table 4" -> "Table 6"?

Table numbering amended.

302-303: "… emphasises the importance of both analysing and interpreting data, and making this information easily accessible to decision-makers" -> I would agree that these are important issues. But I don't see how this paper backs this up. Instead, the paper focuses on which type of impact is generated and by which means this is done (which is already a highly relevant topic). Thus, I would suggest that this sentence is reformulated accordingly (either the second part removed, or some additional arguments given backing up this claim).

This line is referring to the impact pathways aspect of the research. This has now been made explicit in the main text.

I can confirm that all authors have approved the manuscript and agree with its submission to PLOS ONE. I can also confirm that the manuscript is the sole work of the authors and has not been submitted for publication anywhere else.

Thank you again for considering this submission and I look forward to hearing from you.

Sincerely,

Eric Jensen

Paul Wong

Mark Reed
