# Supplementary material for: How research data deliver non-academic impacts: A secondary analysis of UK Research Excellence Framework impact case studies
Source: PLoS One. 2022 Mar 10;17(3):e0264914. doi: 10.1371/journal.pone.0264914 (PMC8912237; doi:10.1371/journal.pone.0264914)
Supplement: Attachment — Submitted filename: Response to Reviewers (Jensen et al.)V2.docx [file pone.0264914.s002.docx]

**Resubmission (rebuttal) Letter**

Response to Reviewers

Editor

PLOS ONE

8 Oct 2021

Dear PLOS ONE Team,

I hope this letter finds you well. I am writing to you on behalf of myself and my co-authors again regarding our manuscript “How research data delivers non-academic impacts: A secondary analysis of UK Research Excellence Framework impact case studies”.

The comments from the reviewers have been pasted in below with responses in blue.

Reviewer #1: Previous literature about non-academic impact in relation to REF should be reviewed more inclusively in the introduction.

As noted previously additional literature has been integrated into the introduction and discussion, focusing on other recent analyses of REF2014 case studies, and the inclusion of impact in REF. We would appreciate the reviewer point us to specific papers or publications that should be included for review.

Table 2 contains main empty rows that probably should be deleted.

Empty rows deleted. Please note Word does not track changes to modification of table structure.

I tried to reproduce the results. I obtain an error: 'inputs/ImpactOnly.csv' does not exist in current working directory'.

The error seems to occur in line 96 of the R script refimpact.R:

ImpactCase <<- read_delim("inputs/ImpactOnly.csv", "\t", escape_double = FALSE, trim_ws = TRUE)

I find only the following files in that directory:

0-GPA.csv CaseStudyID.csv impactid-ukprn.csv UoA.csv

Please check the Git repository.

We very much appreciate the reviewer taking the effort to rerun the R script. Line 96 does reference a file called ImpactOnly.csv. But the omission of ImpactOnly.csv is intentional. The universities in the UK could not agree on a single licensing arrangement. As such many universities retained their rights over their impact cases. While we have obtained permission from the REF UK to use the impact cases for our study here, we do not have the right to make copies and redistribute these cases (see [Term of Use](https://impact.ref.ac.uk/casestudies/Terms.aspx), section on Copies for research). As such, we have not included the file ImpactOnly.csv in the Git repository – the file contains the entire collection of impact description of cases and redistribution of this file could be construed as a violation of the Term of Use. Having said this, we did provide instructions on how to obtain the collection. The README.md file included in the code distribution, section on “[Download the Whole Collection](https://github.com/squarcleconsulting/ukrefimpactmined#download-the-whole-collection)” states:

**By default, refimpact.R assumes that the user has manually downloaded the entire collection and saved the file in ukrefimpact/inputs/ImpactOnly.csv. Follow the instruction below to download the whole collection:**

- **Go to UK Impact Case Studies and click “See all case studies”**
- **Scroll down and click “None selected”**
- **Check “Details of impact” and proceed to click “download”**
- **When download is completed, open CaseStudies.xlsx in Excel**
- **Rename columns: “Case Study Id” with “ID”, “Unit of Assessment” with “FoR” and “Details of the impact” with “impact”**
- **Use TRIM(CLEAN(SUBSTITUTE(cell, CHAR(160), “ “))) in Excel to remove all non-printable characters (including line breaks) and extra white spaces in “impact” column**
- **Remove column “Institution” and “Title”**
- **Save CaseStudies.xlsx as a tab delimited text file with the name “ImpactOnly.csv”**
- **Put ImpactOnly.csv in refimpact/inputs**

**Alternatively, you can turn on automatic download with the fetchUKImpact(auto=TRUE) call. But by default the function will only download the first five impact cases, you can set the value using k=... option.**

Please also note that the README.md also states that the R version tested was 3.4.4 under Ubuntu 16.04.5 and OSX 10.13.6. We cannot guarantee the code will run under a different operating systems or environment.

Reviewer #2: The paper has much improved and all points have been addressed. There are only a few minor points that should be changed/clarified:

Table 1: "in Q3" -> "above Q3"

Amended.

Table 2: This table is much more understandable now, but now the different dimensions for impact type are missing, while the dimensions for impact pathway are still included. Showing the results also for the impact pathway dimensions, or giving a reason why you don't show them, would make this more consistent.

Table 2 has referenced both table 3 and 5. We feel that the inclusion of details from table 3 and 5 inside table 2 would make it more difficult to interpret and understand table 2.

257-258: "Table 5. Identified ways of developing impact, or impact pathways." -> "Table 5."

It is unclear to us why the title of table 5 should be omitted. All the tables in the paper include a short title/description. Such an omission would make it inconsistent with the rest of the paper.

I can confirm that all authors have approved the manuscript and agree with its submission to PLOS ONE. I can also confirm that the manuscript is the sole work of the authors and has not been submitted for publication anywhere else.

Thank you again for considering this submission and I look forward to hearing from you.

Sincerely,

Paul Wong

Eric Jensen

Mark Reed
