# Supplementary material for: How research data deliver non-academic impacts: A secondary analysis of UK Research Excellence Framework impact case studies
Source: PLoS One. 2022 Mar 10;17(3):e0264914. doi: 10.1371/journal.pone.0264914 (PMC8912237; doi:10.1371/journal.pone.0264914)
Supplement: Attachment — Submitted filename: Response to Reviewers (Jensen et al.)V3.docx [file pone.0264914.s003.docx]

**Resubmission (rebuttal) Letter**

Response to Reviewers

Editor

PLOS ONE

24 Nov 2021

Dear PLOS ONE Team,

I hope this letter finds you well. I am writing to you on behalf of myself and my co-authors again regarding our manuscript “How research data delivers non-academic impacts: A secondary analysis of UK Research Excellence Framework impact case studies”.

The comments from the reviewers have been pasted in below with responses in blue.

Reviewer #2: There is only one minor point I would still recommend to change. The title of table 5 should be removed in the text (ll. 257-258), not in the table caption itself (l. 260). All other mentions of tables in the text do not seem to include the titles (e.g. "There are 148 remaining cases as noted in Table 1. A manual review of these 148 cases were conducted to ensure their relevance", ll. 155-157).

The authors can confirm that all references to tables use the designation “Table X” with the title removed.

I can confirm that the reference has been checked for retraction (none found). We have updated the reference because one cited paper has changed status from “in press” to “published”.

I can also confirm that all authors have approved the manuscript and agree with its submission to PLOS ONE. I can also confirm that the manuscript is the sole work of the authors and has not been submitted for publication anywhere else.

Thank you again for considering this submission and I look forward to hearing from you.

Sincerely,

Paul Wong

Eric Jensen

Mark Reed
